# Author Correction: In situ structure of a bacterial flagellar motor at subnanometre resolution reveals adaptations for increased torque

**DOI:** 10.1038/s41564-025-02082-9

**Published:** 2025-07-14

**Authors:** Tina Drobnič, Eli J. Cohen, Thomas Calcraft, Mona Alzheimer, Kathrin Froschauer, Sarah Svensson, William H. Hoffmann, Nanki Singh, Sriram G. Garg, Louie D. Henderson, Trishant R. Umrekar, Andrea Nans, Deborah Ribardo, Francesco Pedaci, Ashley L. Nord, Georg K. A. Hochberg, David R. Hendrixson, Cynthia M. Sharma, Peter B. Rosenthal, Morgan Beeby

**Affiliations:** 1 Department of Life Sciences, Imperial College London, London, UK; 2 Structural Biology of Cells and Viruses Laboratory, The Francis Crick Institute, London, UK; 3 University of Würzburg, Institute of Molecular Infection Biology, Department of Molecular Infection Biology II, Würzburg, Germany; 4 Centre de Biologie Structurale, Universite de Montpellier, CNRS, INSERM, Mont-pellier, France; 5 Max Planck Institute for Terrestrial Microbiology, Marburg, Germany; 6 Structural Biology Science Technology Platform, The Francis Crick Institute, London, UK; 7 Department of Microbiology, University of Texas Southwestern Medical Center, Dallas, TX USA; 8 Department of Biology, Marburg university, Marburg, Germany; 9 Present Address: MRC Laboratory of Molecular Biology, Francis Crick Avenue, Cambridge, UK; 10 Present Address: The Center for Microbes, Development and Health, CAS Key Laboratory of Molecular Virology and Immunology, Shanghai Institute of Immunity and Infection, Chinese Academy of Sciences, Shanghai, China

**Keywords:** Cryoelectron microscopy, Bacterial structural biology, Cryoelectron tomography, Motor protein structure, Molecular evolution

Correction to: *Nature Microbiology* 10.1038/s41564-025-02012-9, published online 1 July 2025.

In the version of the article initially published, the surname of William H. Hoffmann appeared incorrectly (as Hoffman) and has now been amended in the HTML and PDF versions of the article.

