# Supplementary material for: In situ structure of a bacterial flagellar motor at subnanometre resolution reveals adaptations for increased torque
Source: Nat Microbiol. 2025 Jul 1;10(7):1723–40. doi: 10.1038/s41564-025-02012-9 (PMC12222027; doi:10.1038/s41564-025-02012-9)
Supplement: Supplementary file 1 — Reporting Summary [file 41564_2025_2012_MOESM1_ESM.pdf]

nature portfolio

Corresponding author(s): Morgan Beeby

Last updated by author(s): Jan 29, 2025

Reporting Summary

Nature Portfolio wishes to improve the reproducibility of the work that we publish. This form provides structure for consistency and transparency in reporting. For further information on Nature Portfolio policies, see our Editorial Policies and the Editorial Policy Checklist.

Statistics

For all statistical analyses, confirm that the following items are present in the figure legend, table legend, main text, or Methods section.

n/a	Confirmed	
☐	☒	The exact sample size (*n*) for each experimental group/condition, given as a discrete number and unit of measurement
☒	☐	A statement on whether measurements were taken from distinct samples or whether the same sample was measured repeatedly
☒	☐	The statistical test(s) used AND whether they are one- or two-sided<br>*Only common tests should be described solely by name; describe more complex techniques in the Methods section.*
☒	☐	A description of all covariates tested
☒	☐	A description of any assumptions or corrections, such as tests of normality and adjustment for multiple comparisons
☒	☐	A full description of the statistical parameters including central tendency (e.g. means) or other basic estimates (e.g. regression coefficient) AND variation (e.g. standard deviation) or associated estimates of uncertainty (e.g. confidence intervals)
☒	☐	For null hypothesis testing, the test statistic (e.g. *F*, *t*, *r*) with confidence intervals, effect sizes, degrees of freedom and *P* value noted<br>*Give P values as exact values whenever suitable.*
☒	☐	For Bayesian analysis, information on the choice of priors and Markov chain Monte Carlo settings
☒	☐	For hierarchical and complex designs, identification of the appropriate level for tests and full reporting of outcomes
☒	☐	Estimates of effect sizes (e.g. Cohen's *d*, Pearson's *r*), indicating how they were calculated

Our web collection on statistics for biologists contains articles on many of the points above.

Software and code

Policy information about availability of computer code

Data collection	All data collection software is fully described in Methods. In brief, single particle analysis data was acquired using EPU (Thermo Fisher Scientific). Tomography data was acquired with either Tomo5 (Thermo Fisher Scientific) or Leginon (developed by an independent group, cited appropriately).
Data analysis	All data analysis software is fully described in Methods. In brief, single particle analysis was performed entirely using RELION 3.1 (cited appropriately in Methods). Subtomogram averaging was performed in IMOD and PEET (cited appropriately).

For manuscripts utilizing custom algorithms or software that are central to the research but not yet described in published literature, software must be made available to editors and reviewers. We strongly encourage code deposition in a community repository (e.g. GitHub). See the Nature Portfolio guidelines for submitting code & software for further information.

Data

Policy information about availability of data

All manuscripts must include a data availability statement. This statement should provide the following information, where applicable:

- Accession codes, unique identifiers, or web links for publicly available datasets
- A description of any restrictions on data availability
- For clinical datasets or third party data, please ensure that the statement adheres to our policy

Cryo-EM maps have been deposited at the Electron Microscopy Data Bank (EMDB) and are publicly available as of the date of publication. The whole-motor map

has been deposited with accession code EMD-16723 together with the original micrographs deposited to the EMPIAR repository with public accession code EMPIAR-11580 (DOI: 10.6019/EMPIAR-10016). The refined periplasmic scaffold map has been deposited in the EMDB with accession code EMD-16724. Subtomogram average maps were deposited as follows: ΔpflC - EMD-17415; ΔpflD - EMD-17416; pflAΔ16-168 - EMD-17417; FlgQ-mCherry - EMD-17419. The α-carbon coordinates of the atomic model of the periplasmic scaffold has been deposited to the PDB with public accession code 9HMF.

Research involving human participants, their data, or biological material

Policy information about studies with human participants or human data. See also policy information about sex, gender (identity/presentation), and sexual orientation and race, ethnicity and racism.

Reporting on sex and gender	N/A
Reporting on race, ethnicity, or other socially relevant groupings	N/A
Population characteristics	N/A
Recruitment	N/A
Ethics oversight	N/A

Note that full information on the approval of the study protocol must also be provided in the manuscript.

Field-specific reporting

Please select the one below that is the best fit for your research. If you are not sure, read the appropriate sections before making your selection.

☒ Life sciences ☐ Behavioural & social sciences ☐ Ecological, evolutionary & environmental sciences

For a reference copy of the document with all sections, see nature.com/documents/nr-reporting-summary-flat.pdf

Life sciences study design

All studies must disclose on these points even when the disclosure is negative.

Sample size	We acquired approximately 95,000 particles for single particle analysis limited primarily by microscope access time.
Data exclusions	Particles were rejected during 2-D and 3-D classification as per field conventions as outlined in Extended Data Figure 1.
Replication	All statistics are calculated by splitting the dataset into two halves as per field convention of 'Gold Standard Fourier Shell Coefficient' as reported in Methods.
Randomization	N/A
Blinding	N/A

Reporting for specific materials, systems and methods

We require information from authors about some types of materials, experimental systems and methods used in many studies. Here, indicate whether each material, system or method listed is relevant to your study. If you are not sure if a list item applies to your research, read the appropriate section before selecting a response.

Materials & experimental systems

n/a	Involved in the study	
☐	☒	Antibodies
☒	☐	Eukaryotic cell lines
☒	☐	Palaeontology and archaeology
☒	☐	Animals and other organisms
☒	☐	Clinical data
☒	☐	Dual use research of concern
☒	☐	Plants

Methods

n/a	Involved in the study	
☒	☐	ChIP-seq
☒	☐	Flow cytometry
☒	☐	MRI-based neuroimaging

Antibodies

Antibodies used	FlgP specific antisera Ref21 FlgP specific antisera<br>Anti-GFP Roche #11814460001, RRID:AB_390913<br>Anti-FLAG Sigma-Aldrich #F1804-1MG, RRID:AB_262044<br>Anti-mouse IgG GEHealthcare #RPN4201<br>Anti-rabbit IgG GEHealthcare #RPN4301
Validation	As per above for the four commercial antibodies. FlgP specific antisera derives from Sommerlad, S. M. & Hendrixson, D. R. "Analysis of the roles of FlgP and FlgQ in flagellar motility of Campylobacter jejuni." J. Bacteriol 189, 179–186 (2007), in which wild-type and FlgP-deficient strains as positive and negative controls demonstrate positive and negative responses to the antisera respectively.

Plants

Seed stocks	N/A
Novel plant genotypes	N/A
Authentication	N/A
