# Supplementary material for: In situ structure of a bacterial flagellar motor at subnanometre resolution reveals adaptations for increased torque
Source: Nat Microbiol. 2025 Jul 1;10(7):1723–40. doi: 10.1038/s41564-025-02012-9 (PMC12222027; doi:10.1038/s41564-025-02012-9)

Raw images of gels from top row of Fig. 3E. PflC-2xFLAG and FlgP CoIP:

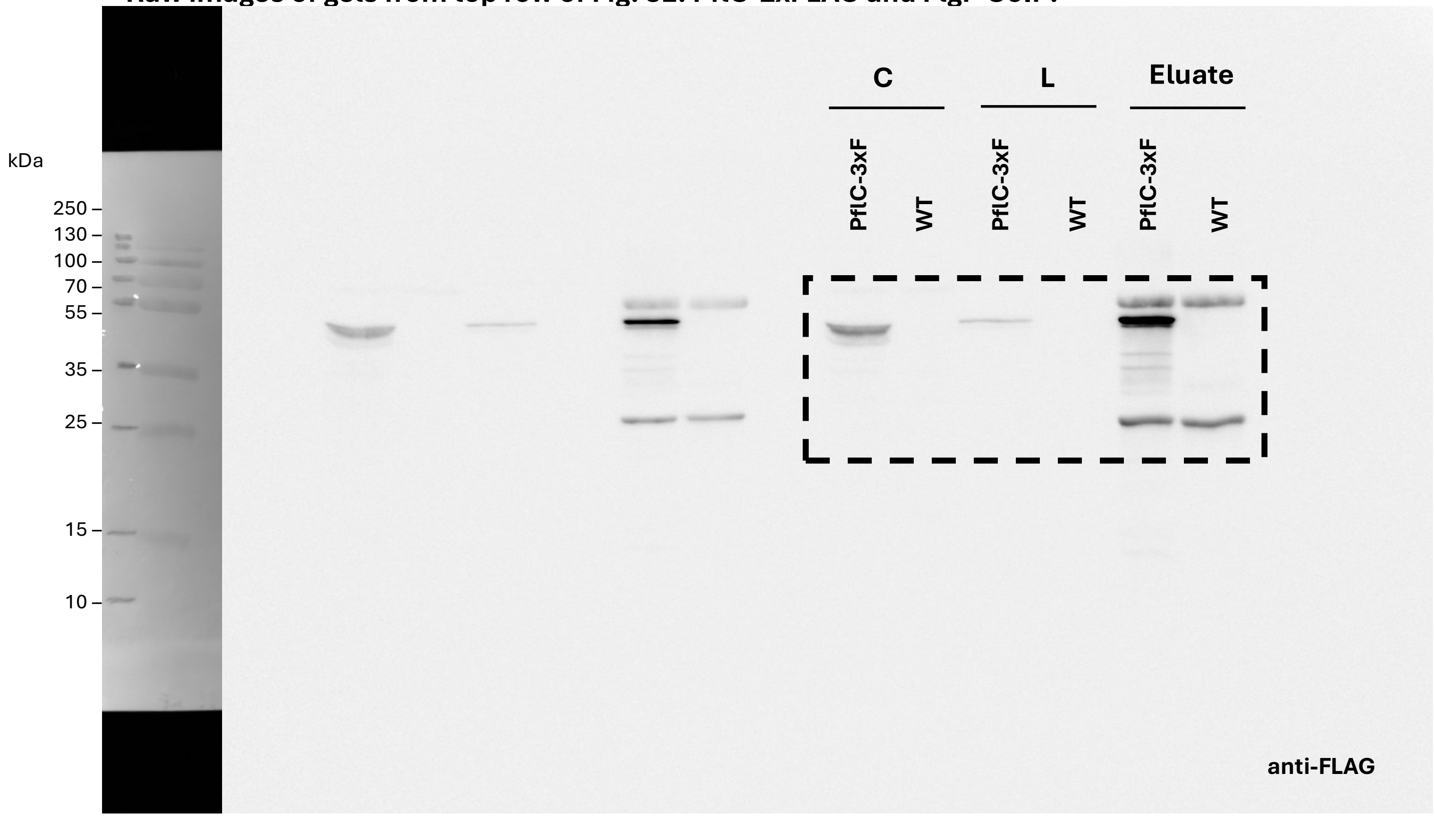

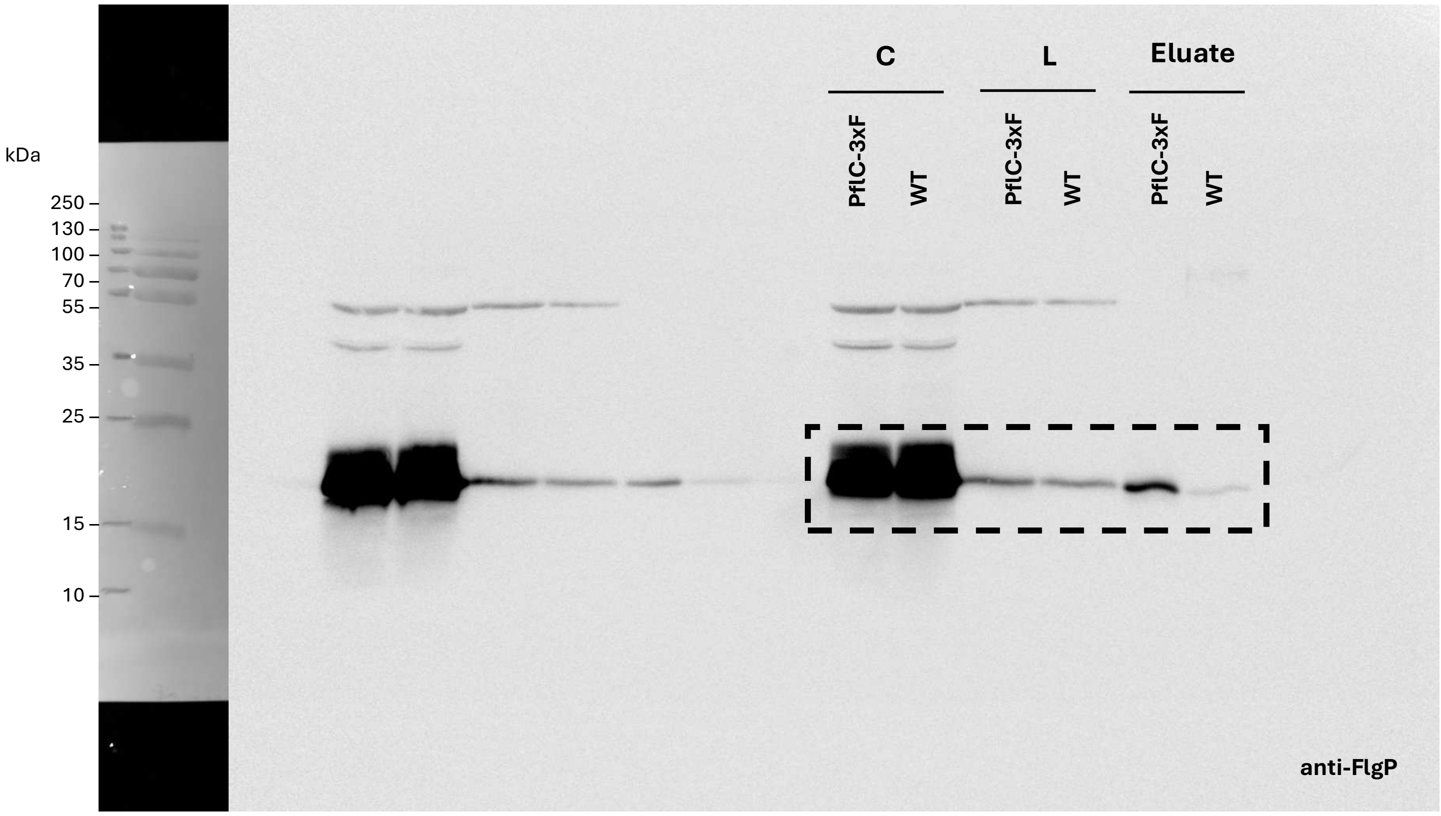

Raw images of gels from middle row of Fig. 3E. PflA and PflD CoIP:

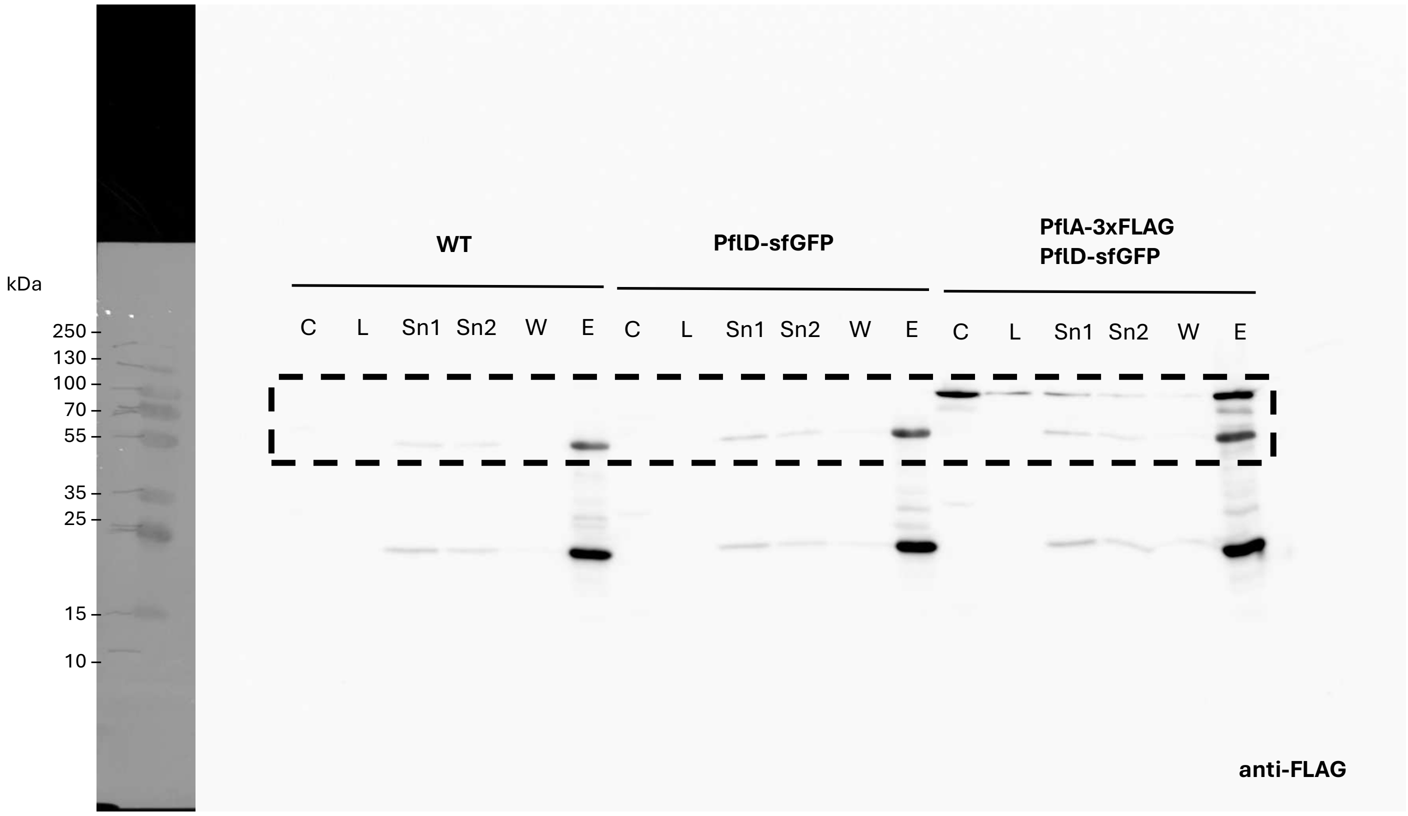

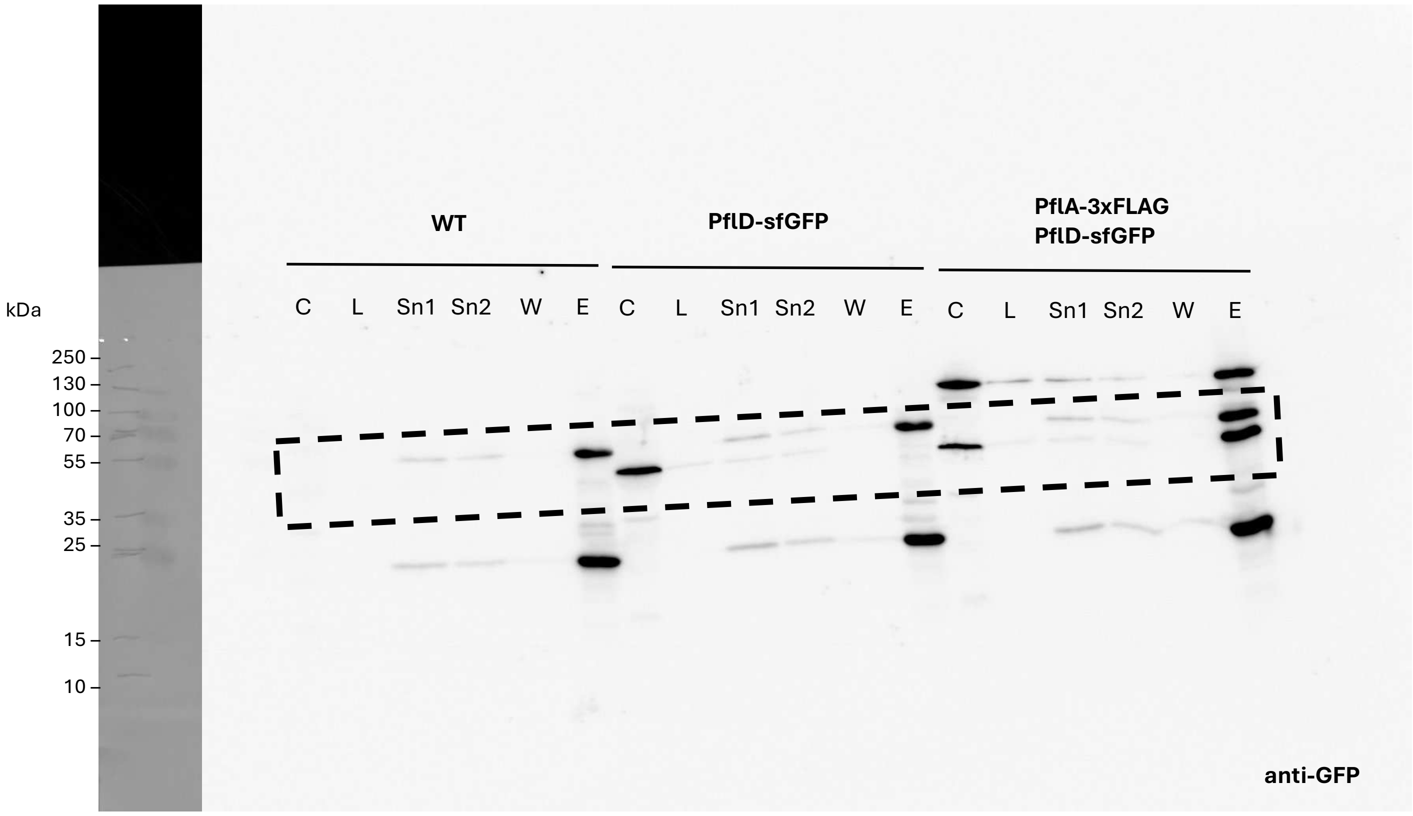

Raw images of gels from bottom row of Fig. 3E. PflB-3xFLAG and PflD-sfGFP CoIP:

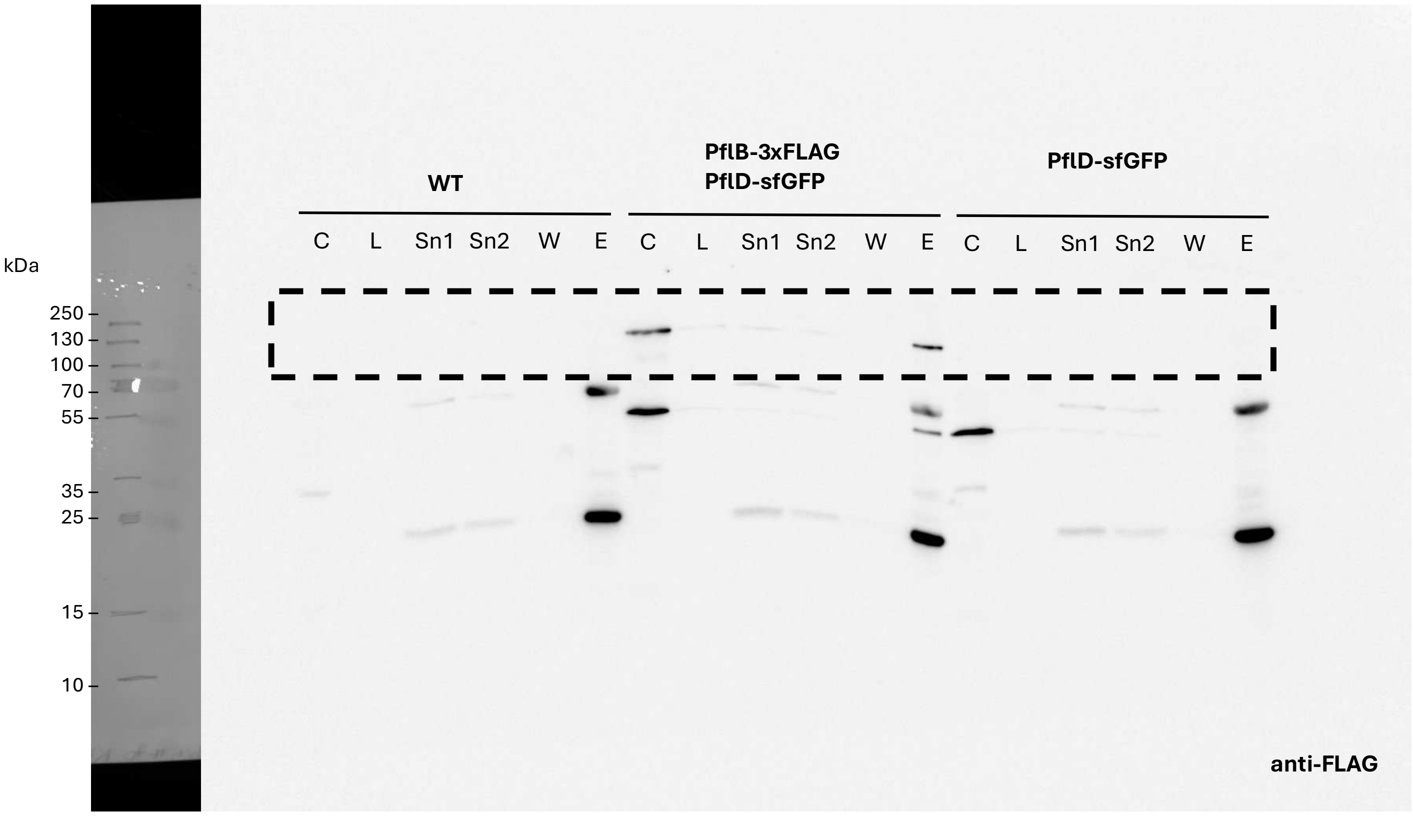

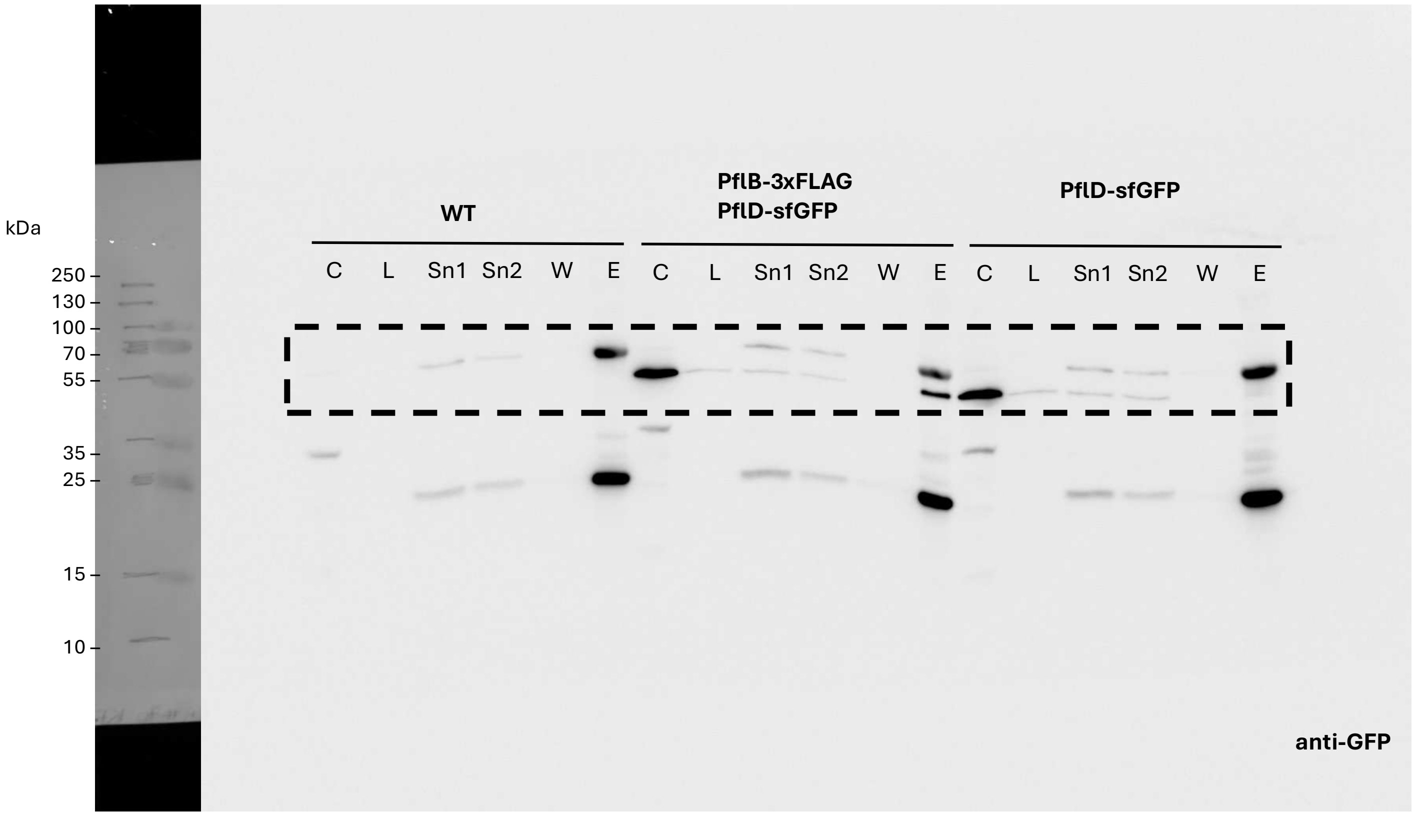

Supplement: Supplementary file 4 — Unprocessed gels for Fig. 3e. [file 41564_2025_2012_MOESM4_ESM.pdf]
